# Postoperative Delirium After OLIF-PPSF Versus TLIF: A Propensity Score-Matched Analysis over 16 Years

**DOI:** 10.3390/jcm15207930

**Published:** 2026-10-14

**Authors:** Myung-Jin Sung, Yoon-Koo Kim, Sung-Kyu Kim

**Affiliations:** Department of Orthopedic Surgery, Chonnam National University Medical School and Hospital, Gwangju 61469, Republic of Korea; smj2383@naver.com (M.-J.S.); osthing@naver.com (Y.-K.K.)

**Keywords:** oblique lateral interbody fusion, percutaneous pedicle screw fixation, transforaminal lumbar interbody fusion, delirium, transfusion, operative time

## Abstract

**Background/Objectives:** Postoperative delirium is a clinically important complication after lumbar fusion surgery, yet whether the choice of fusion technique influences its occurrence remains unclear. We compared the incidence of postoperative delirium between oblique lateral interbody fusion with percutaneous pedicle screw fixation (OLIF-PPSF) and transforaminal lumbar interbody fusion (TLIF). **Methods:** The medical records of 850 patients (335 OLIF-PPSF, 515 TLIF) treated between 2008 and 2023 were retrospectively reviewed; 487 patients were eligible, and propensity score matching yielded 141 matched pairs. Delirium was identified through symptom-triggered psychiatric consultation rather than routine screening. Analyses were stratified by treatment era and repeated within the period in which both procedures were performed. **Results:** Delirium occurred in no OLIF-PPSF patient and in 9 TLIF patients (6.4%; risk difference 6.4%, 95% CI 2.4–11.7%; McNemar exact *p* = 0.004; adjusted odds ratio 19.82, 95% CI 1.20–326.53). However, OLIF-PPSF was performed only from 2017 onward, whereas 118 of the 141 matched TLIF operations (83.7%) preceded 2017, and all events occurred during 2008–2014. Within 2017–2023 no delirium occurred after either procedure (0/168 versus 0/34). **Conclusions:** Although delirium was less frequent after OLIF-PPSF, every event preceded its introduction, and none occurred after either procedure once both were performed contemporaneously. The difference cannot be attributed to the surgical approach and more likely reflects changes in perioperative care over time. These findings are exploratory.

## 1. Introduction

Lumbar interbody fusion has become a widely accepted surgical treatment for degenerative lumbar disorders causing chronic low back pain, radiculopathy, and functional disability refractory to conservative care [1,2]. Conventional techniques, including anterior, posterior, and transforaminal lumbar interbody fusion (TLIF), achieve reliable fusion and satisfactory clinical outcomes [3]. However, these open posterior approaches require extensive paraspinal muscle dissection, prolonged operative time, and considerable intraoperative blood loss, which may increase perioperative morbidity [4]. Unlike surgery of the extremities, where a tourniquet can produce a bloodless operative field, spinal fusion does not allow tourniquet use and is performed within a highly vascular region rich in paravertebral musculature and epidural venous plexuses; consequently, intraoperative blood loss is often substantial. As the population undergoing lumbar fusion continues to age, minimizing perioperative complications has become increasingly important.

To reduce approach-related morbidity, muscle-sparing minimally invasive techniques have been developed. Oblique lateral interbody fusion (OLIF), an increasingly adopted muscle-sparing technique typically combined with percutaneous pedicle screw fixation (PPSF), accesses the disc space through an oblique retroperitoneal corridor, preserving the lamina, facet joints, and paraspinal musculature while achieving indirect decompression and stable fusion [5,6]. Compared with TLIF, OLIF-PPSF has been reported to reduce blood loss, shorten operative time, and lessen tissue injury while providing comparable clinical and radiographic outcomes [7,8,9]. Together, TLIF and OLIF-PPSF are now among the most widely used interbody fusion techniques for degenerative lumbar disease [3,5]. In parallel, endoscopic variants of the transforaminal approach (endoscopic TLIF) have recently gained increasing popularity as a further minimally invasive refinement [10].

Postoperative delirium is a clinically significant but underrecognized complication after spinal surgery, particularly in older adults. Its reported incidence after lumbar fusion ranges widely, from approximately 3% to more than 20%, with higher rates in elderly patients and after more invasive procedures [11,12]. Delirium is associated with prolonged hospitalization, functional decline, falls, increased medical cost, and higher mortality [13,14]. Advanced age, prolonged operative time, blood loss, transfusion, and postoperative opioid exposure have been identified as contributors to delirium after major surgery, including spinal procedures [15,16], plausibly through systemic inflammation, cerebral hypoperfusion, and blood–brain barrier dysfunction [17]. Because these factors differ with surgical invasiveness, the fusion technique may influence delirium risk; nevertheless, despite extensive comparisons of radiographic and functional outcomes, the incidence of postoperative delirium after OLIF-PPSF versus TLIF has not been specifically investigated.

Therefore, we compared the incidence of postoperative delirium between patients undergoing OLIF-PPSF and those undergoing TLIF using propensity score-matched cohorts. We hypothesized that OLIF-PPSF would be associated with a lower incidence of delirium than TLIF, owing to its reduced surgical invasiveness, particularly shorter operative time and lower transfusion requirements. Because OLIF-PPSF was introduced at our institution in 2017 whereas TLIF had been performed since 2008, we also examined the calendar distribution of the two procedures and the temporal trends in delirium and transfusion.

## 2. Materials and Methods

### 2.1. Study Design and Patient Selection

**Figure 1:**
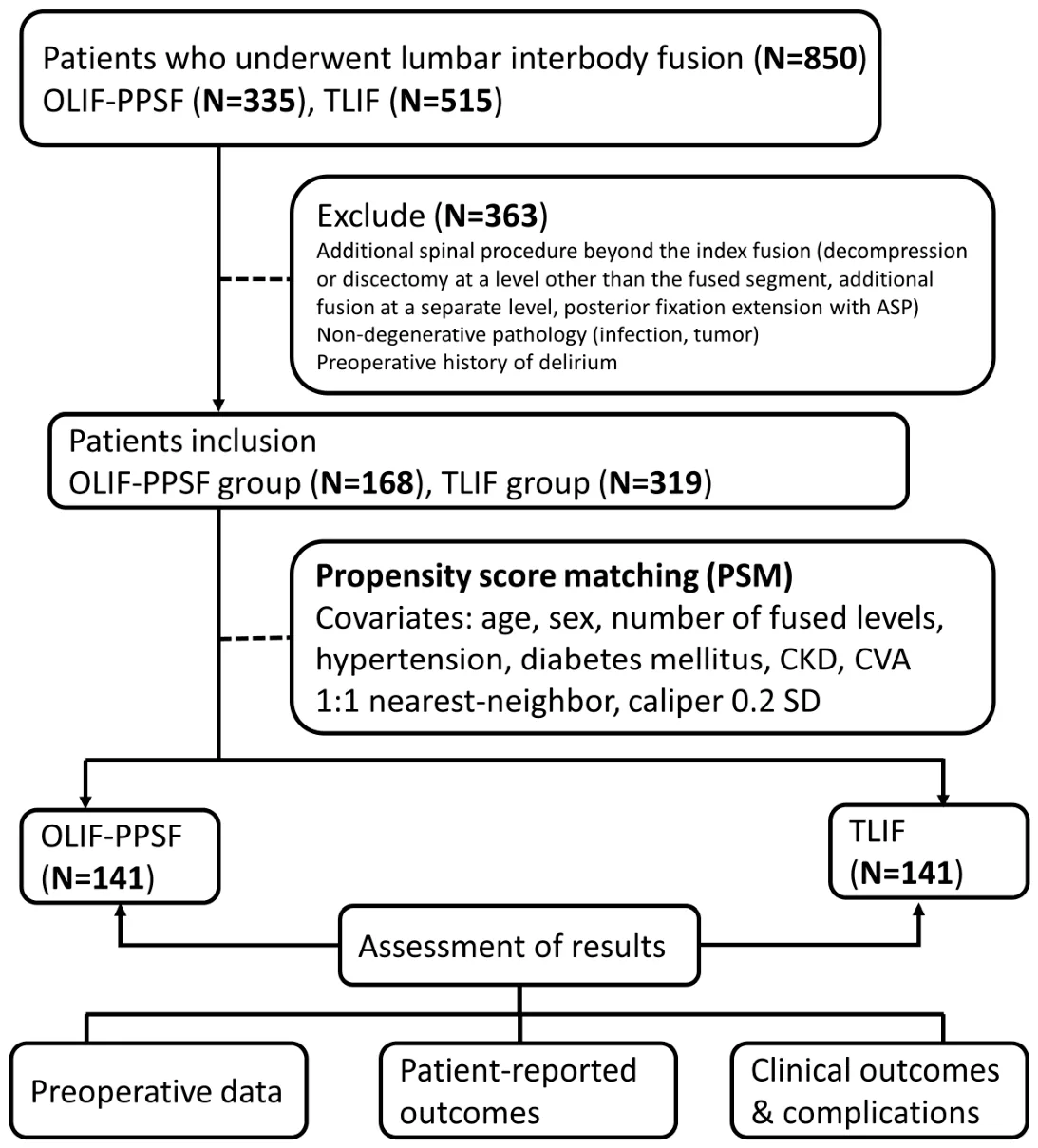
Flow chart of patient inclusion and assessment. OLIF-PPSF, oblique lateral interbody fusion with percutaneous pedicle screw fixation; TLIF, transforaminal lumbar interbody fusion; ASP, adjacent-segment plate; PSM, propensity score matching; CKD, chronic kidney disease; CVA, cerebrovascular accident; SD, standard deviation.

This retrospective comparative study was approved by the institutional review board of Chonnam National University Hospital (approval no. CNUH-2026-226), which waived the requirement for informed consent. The medical records of all patients who underwent OLIF-PPSF or TLIF for degenerative lumbar disease at our institution between 2008 and 2023 were reviewed. All OLIF-PPSF and TLIF procedures were performed by a single senior spine surgeon at this single institution. All TLIF procedures were minimally invasive TLIF performed through a paraspinal tubular approach with unilateral facetectomy for direct neural decompression, and OLIF-PPSF was performed through an oblique retroperitoneal corridor to achieve indirect decompression. A polyetheretherketone cage was used in both procedures, filled with allograft in OLIF-PPSF and with a mixture of local autograft and allograft in TLIF. Intraoperative navigation was not used, and percutaneous pedicle screw fixation was performed under C-arm fluoroscopic guidance in both groups. For OLIF-PPSF, the cage was inserted with the patient in the lateral decubitus position, after which the patient was repositioned prone for posterior fixation. All procedures were performed under general anesthesia. OLIF-PPSF was introduced at our institution in 2017; before that year, all interbody fusions in this series were performed as TLIF. In general, TLIF was selected when direct neural decompression was considered necessary—for example, for a sequestered or migrated disc herniation or high-grade central stenosis—or for L5–S1 pathology, where the iliac crest and iliac vessels limit the oblique retroperitoneal corridor; OLIF-PPSF was otherwise selected for degenerative pathology amenable to indirect decompression. The initial cohort comprised 850 patients (335 OLIF-PPSF, 515 TLIF). Patients were excluded if they underwent a concurrent additional spinal procedure beyond the index fusion (decompression or discectomy at a level other than the fused segment, an additional fusion at a separate, non-contiguous level, or extension of posterior fixation with an adjacent-segment plate), if the surgery was performed for a non-degenerative condition such as infection or tumor, or if they had a preoperative history of delirium. After exclusions, 487 patients (168 OLIF-PPSF, 319 TLIF) were eligible for analysis (Figure 1). The unit of analysis was the operation; seven patients contributed two eligible operations each. All included patients had a minimum postoperative follow-up of 12 months, ensuring that patient-reported outcomes were assessed at a comparable and clinically mature time point.

To reduce selection bias arising from baseline differences between the groups, propensity score matching (PSM) was performed. Propensity scores were estimated from a multivariable logistic regression model that included age, sex, number of fused levels, hypertension, diabetes mellitus, chronic kidney disease, and cerebrovascular accident; these covariates were selected a priori on the basis of their clinical relevance to postoperative delirium and their imbalance between the groups before matching. One-to-one nearest-neighbor matching without replacement was applied using a caliper of 0.2 standard deviations of the logit of the propensity score. Covariate balance was assessed using standardized mean differences (SMDs), with an absolute SMD below 0.10 regarded as good balance and below 0.20 as acceptable [18]. Matching yielded 141 matched pairs, which constituted the analytic cohort for all subsequent comparisons. Dementia, although a recognized risk factor for postoperative delirium, was already well balanced between the groups before matching (standardized mean difference 0.04) and was therefore not included as a matching covariate; its balance was preserved after matching, and it was examined separately in the risk-factor analysis. We recognize that a covariate may be prognostic even when balanced, but the small number of events did not permit a larger matching model. Calendar year was deliberately not included in the propensity score model, because no OLIF-PPSF procedure was performed before 2017 and the positivity assumption required for such adjustment was therefore not met [19].

### 2.2. Perioperative Data and Outcomes

Patient demographics (age, sex, body mass index), underlying diagnosis, comorbidities (hypertension, diabetes mellitus, hepatitis, chronic kidney disease, cerebrovascular accident, and dementia), and surgical parameters (number of fused levels, operative time, estimated blood loss, transfusion, and length of hospital stay) were extracted from the medical records. Estimated blood loss was obtained from the intraoperative anesthesia records as documented by the attending anesthesiologist; a formula-based calculation was not used. Transfusion was defined as any allogeneic red blood cell transfusion administered intraoperatively or during the postoperative hospital stay, and was given when the hemoglobin concentration fell below 8 g/dL, the same threshold being applied throughout the study period [20]. The primary outcome was in-hospital postoperative delirium; delirium occurring after discharge was not captured.

The diagnosis of postoperative delirium was established by a consulting psychiatrist according to the criteria of the Diagnostic and Statistical Manual of Mental Disorders in force at the time (DSM-IV-TR before 2013 and DSM-5 thereafter) [21]. Psychiatric consultation was requested whenever signs suggestive of delirium—disorientation, memory impairment, emotional or perceptual disturbance, or psychomotor change—were observed during the postoperative hospital stay. Symptoms were identified by ward nurses and physicians during routine care rather than at fixed assessment intervals, and every patient in whom delirium was suspected was referred to the Department of Psychiatry; the consulting psychiatrist was aware of the operation performed and was therefore not blinded. The records were not retrospectively re-screened with a validated chart-based instrument. This ascertainment process was unchanged throughout the study period, and no routine screening instrument and no delirium-prevention protocol were introduced. The day of onset was defined as the postoperative day on which psychiatric consultation was first requested; postoperative delirium typically develops within the first five postoperative days [22]. Because opioids and benzodiazepines are recognized precipitants of delirium [15,16], their postoperative use (administration at least once during hospitalization, regardless of dose) was also recorded.

### 2.3. Assessment of Patient-Reported Outcomes

Patient-reported outcomes were assessed using the visual analog scale (VAS) for pain, the Japanese Orthopaedic Association (JOA) score for low back pain (range −6 to 29, with higher scores indicating better function) [23], and the Oswestry Disability Index (ODI), measured one day before surgery and at the last follow-up (a minimum of 12 months postoperatively).

### 2.4. Statistical Analysis

Continuous variables are presented as mean ± standard deviation and categorical variables as number (percentage). For the matched cohort, the delirium comparison was made with McNemar’s exact test, and the absolute risk difference is reported with a 95% confidence interval calculated by Newcombe’s hybrid score method [24]; Fisher’s exact test is also reported. Because matched-pair identifiers were not retained in the matching output, continuous variables were compared with independent-sample *t*-tests rather than paired tests [25]. The estimand was the average treatment effect in the matched population.

Because no delirium occurred in the OLIF-PPSF group, risk factors were analyzed using Firth’s penalized-likelihood logistic regression, which yields finite estimates under quasi-complete separation [26,27]. Given the small number of events, the multivariable model was limited to surgical group and age to estimate the total effect of the surgical approach; transfusion, which lies on the causal pathway between approach and delirium, was instead assessed in a separate model, which is reported as exploratory because formal estimation of direct and indirect effects was not feasible with so few events [28]. An exploratory age threshold was derived from the receiver operating characteristic (ROC) curve using the Youden index [29] in the eligible TLIF cohort, in which more events were available. Analyses were performed using SPSS version 26.0 (IBM Corp., Armonk, NY, USA), with Firth regression performed in R version 4.3 (R Foundation for Statistical Computing, Vienna, Austria) using the logistf package version 1.26.0; a two-sided *p* < 0.05 was considered statistically significant. To examine the influence of calendar time, the distribution of operations by year was tabulated, delirium and transfusion rates were compared across four predefined eras (2008–2011, 2012–2014, 2015–2016, and 2017–2023), and the comparison between the procedures was repeated after restriction to 2017–2023. A sensitivity analysis excluded matched pairs that contained a patient’s second operation.

## 3. Results

### 3.1. Patient Cohort and Temporal Distribution of the Two Procedures

After application of the exclusion criteria, 487 patients (168 OLIF-PPSF, 319 TLIF) were eligible, and propensity score matching yielded 141 matched pairs (Figure 1). The mean duration of follow-up for patient-reported outcomes was 40.3 months in the OLIF-PPSF group and 41.7 months in the TLIF group (*p* = 0.673); the observation window for delirium was the index hospital admission.

The two procedures occupied almost non-overlapping calendar periods. OLIF-PPSF was performed only between 2017 and 2023, whereas 118 of the 141 matched TLIF operations (83.7%) were performed before 2017; only 23 matched TLIF operations (16.3%) fell within the period in which OLIF-PPSF was also being performed (Table 1).

### 3.2. Baseline Characteristics

Before matching, the two groups differed substantially in several baseline characteristics: patients in the OLIF-PPSF group were older (69.5 vs. 64.4 years) and had a higher number of fused levels and a greater prevalence of hypertension, chronic kidney disease, and cerebrovascular accident, with standardized mean differences exceeding 0.20 for each. After matching, all seven covariates included in the propensity score model had standardized mean differences below 0.20 (for example, age, 68.5 vs. 69.0 years; standardized mean difference −0.07), although sex (0.13) and the number of fused levels (0.12) exceeded the 0.10 threshold for good balance. Dementia, although not a matching covariate, was infrequent and comparably distributed between the groups (3.5% vs. 2.8%). The JOA score, which was likewise not included in the propensity score model, showed residual imbalance after matching (14.6 vs. 16.0; standardized mean difference −0.31), whereas the remaining patient-reported scores were balanced. The underlying diagnosis was spinal stenosis in 84 (59.6%) versus 77 (54.6%) and degenerative spondylolisthesis in 57 (40.4%) versus 64 (45.4%) after matching (SMD 0.10), the two groups having been closely comparable in this respect before matching (SMD −0.03), and the distribution of operated levels is also shown in Table 2. Calendar year of surgery, by contrast, remained markedly imbalanced after matching (SMD 2.46). The estimated propensity score ranged from 0.085 to 0.831 in the OLIF-PPSF group and from 0.031 to 0.889 in the TLIF group; the region of common support was 0.085 to 0.831, and 10 of the 487 eligible operations (2.1%) lay outside it. Twenty-seven OLIF-PPSF operations could not be matched because no TLIF counterpart was available within the caliper, and 178 TLIF operations remained unmatched because matching was one-to-one and the TLIF pool was larger. Baseline characteristics before and after matching are presented in Table 2.

### 3.3. Perioperative Outcomes and Postoperative Delirium

Operative time was significantly shorter in the OLIF-PPSF group than in the TLIF group (262.2 ± 75.5 vs. 280.9 ± 66.5 min, *p* = 0.028), and estimated blood loss was lower (283.3 ± 202.3 vs. 393.3 ± 241.1 mL, *p* < 0.001). Transfusion was required far less frequently in the OLIF-PPSF group (20 (14.2%) vs. 96 (68.1%), *p* < 0.001). Postoperative delirium did not occur in any patient in the OLIF-PPSF group, compared with 9 patients (6.4%) in the TLIF group (risk difference 6.4%, 95% CI 2.4–11.7%; McNemar exact *p* = 0.004; Fisher’s exact *p* = 0.003); the incidence was 0% (95% CI 0–2.6%) after OLIF-PPSF and 6.4% (95% CI 3.0–11.8%) after TLIF. Delirium was first diagnosed a median of 3 days after surgery (range 2–17). Among the nine affected patients, the median age was 72 years (range 65–81), five were women, two had preexisting dementia, and seven had received a transfusion; delirium subtype, duration, and treatment were not consistently documented in the medical records. Postoperative opioid and benzodiazepine use did not differ significantly between the groups (opioid, 63 (44.7%) vs. 76 (54.0%), *p* = 0.122; benzodiazepine, 2 (1.4%) vs. 3 (2.1%), *p* = 1.000). The length of hospital stay was longer in the TLIF group (12.3 ± 6.5 vs. 17.7 ± 8.5 days, *p* < 0.001) (Table 3).

### 3.4. Temporal Trends and the Contemporaneous Comparison

All nine delirium events in the matched cohort occurred between 2008 and 2014, that is, before OLIF-PPSF was introduced. In the eligible cohort, the incidence of delirium after TLIF fell from 2.8% in 2008–2011 and 9.0% in 2012–2014 to 2.3% in 2015–2016 and 0% in 2017–2023, and the transfusion rate fell in parallel from 79.6% and 76.9% to 58.1% and 20.6% (Table 4). Both declines therefore began before OLIF-PPSF was introduced. When the comparison was restricted to 2017–2023, no delirium occurred after either procedure (0 of 168 OLIF-PPSF versus 0 of 34 TLIF), and neither the transfusion rate (16.1% versus 20.6%, *p* = 0.615) nor the operative time (268 ± 83 versus 259 ± 56 min, *p* = 0.547) differed significantly between them.

### 3.5. Patient-Reported Outcomes

Postoperative patient-reported outcomes and the degree of improvement from the preoperative assessment did not differ significantly between the two groups for any measure, including the visual analog scale for back and leg pain, the JOA score, and the Oswestry Disability Index. The improvement in the JOA score was somewhat greater after OLIF-PPSF but did not reach significance (3.1 vs. 2.1, *p* = 0.065) (Table 5).

### 3.6. Risk Factors for Postoperative Delirium

Because postoperative delirium did not occur in the OLIF-PPSF group, risk factors were analyzed using Firth’s penalized logistic regression. In the univariable analysis, the TLIF approach (odds ratio 20.30, 95% CI 1.16–355.88, *p* = 0.039) and transfusion (odds ratio 4.52, 95% CI 1.05–19.40, *p* = 0.042) were significantly associated with postoperative delirium, and older age showed a borderline association (odds ratio 1.10 per year, 95% CI 0.99–1.21, *p* = 0.069). In the multivariable model adjusting for age, the TLIF approach remained associated with delirium (adjusted odds ratio 19.82, 95% CI 1.20–326.53, *p* = 0.037), while age retained a borderline association (adjusted odds ratio 1.09 per year, 95% CI 0.99–1.21, *p* = 0.074). Dementia was also associated with delirium (odds ratio 11.84, 95% CI 2.22–63.22, *p* = 0.004), although only two affected patients developed it (Table 6). Because of the temporal separation between the procedures, the group variable cannot be distinguished from calendar period, and these estimates should not be read as an effect of the surgical approach.

When transfusion was entered into the model in place of age, the association of the TLIF approach with delirium was attenuated and no longer reached statistical significance (adjusted odds ratio 16.34, 95% CI 0.89–301.60, *p* = 0.060), and transfusion itself was not independently associated with delirium (adjusted odds ratio 1.49, 95% CI 0.35–6.45, *p* = 0.590) (Table 7). Within the eligible TLIF cohort, in which 16 events were available, older age remained associated with delirium after adjustment for transfusion and era (odds ratio 1.12 per year, 95% CI 1.05–1.20, *p* = 0.001), whereas transfusion did not (odds ratio 0.74, 95% CI 0.23–2.40, *p* = 0.621). The earlier era (before 2015) was itself associated with delirium (odds ratio 6.21, 95% CI 1.05–36.67, *p* = 0.044).

On receiver operating characteristic analysis, age discriminated postoperative delirium with an area under the curve of 0.727, and the exploratory threshold identified by the Youden index was 71 years (sensitivity 69%, specificity 75%); this analysis was performed in the eligible TLIF cohort and was not internally validated.

### 3.7. Sensitivity Analysis

Excluding the four matched pairs that contained a patient’s second operation did not materially change the primary result (0 of 140 versus 9 of 138; odds ratio 20.61, 95% CI 1.18–361.41, *p* = 0.038).

## 4. Discussion

To our knowledge, this is the first study to directly compare the incidence of postoperative delirium between OLIF-PPSF and TLIF using propensity score-matched cohorts. Although OLIF and TLIF have been extensively compared with respect to radiographic alignment, fusion rates, and functional outcomes, previous studies have generally reported overall or approach-related complication rates without specifically examining postoperative delirium [7,8,9,30,31,32,33]. In the present matched cohort, postoperative delirium occurred in 6.4% of TLIF patients but in no OLIF-PPSF patient, and the TLIF approach remained associated with delirium after adjustment for age. The two procedures were, however, performed in almost entirely different calendar periods, and this shaped the comparison in ways considered below.

These findings were accompanied by clear perioperative differences favoring OLIF-PPSF, which had a shorter operative time, lower estimated blood loss, and a markedly lower transfusion requirement. This is consistent with prior comparative studies and meta-analyses reporting that OLIF reduces operative time and intraoperative blood loss relative to TLIF [7,8,9]. Even when performed through a tubular retractor, TLIF requires facetectomy and posterior work within the paraspinal muscles, whereas the retroperitoneal corridor used in OLIF-PPSF avoids the posterior approach altogether. These perioperative differences were not, however, reproduced when the two procedures were compared within the same calendar period, as described below.

This temporal separation was not incidental but reflected a change in institutional practice: after OLIF-PPSF was adopted it became the preferred approach for pathology amenable to indirect decompression, and TLIF was thereafter reserved mainly for cases requiring direct decompression or L5–S1 fusion. Evaluating a new operation against an established one is recognized to be methodologically difficult, in part because adoption is progressive and the choice of comparator is itself problematic [34]; comparisons relying on historical controls have long been recognized to favor the newer treatment [35]. Because no OLIF-PPSF procedure was performed before 2017, calendar time cannot be adjusted for, and the positivity assumption underlying such adjustment is violated [19]. Several observations indicate that the era, rather than the approach, accounts for most of the difference: within the TLIF group alone the incidence of delirium fell from 9.0% in 2012–2014 to 0% after 2017 and the transfusion rate from 76.9% to 20.6%, both declines beginning before OLIF-PPSF was introduced; the length of hospital stay was likewise longer in the TLIF group (17.7 versus 12.3 days); and in the contemporaneous period delirium did not occur after either procedure, with transfusion and operative time no longer differing between them, although the small number of contemporaneous TLIF operations (*n* = 34) limits the precision of that comparison. That comparison should not, however, be read as evidence of equivalence: with only 34 TLIF operations and no events in either group during 2017–2023, it had essentially no power to detect a difference between the procedures.

Postoperative delirium is multifactorial, and advanced age, prolonged operative time, intraoperative blood loss, and transfusion are among its most consistently reported contributors. In a systematic review and meta-analysis of 40 studies, Wu et al. found that the risk of delirium increased with each year of age (OR 1.07) and rose markedly when age exceeded 70 years (OR 15.87) [12]. Pooling studies of spinal surgery, Shi et al. likewise identified prolonged operative duration and greater intraoperative blood loss as significant risk factors [36], consistent with the earlier observations of Gao et al. in patients undergoing spinal surgery [15]. With respect to transfusion specifically, Raphael et al., applying inverse-probability-of-treatment weighting in hip fracture patients, reported that red blood cell transfusion was associated with an increased risk of postoperative delirium [37]. Our results are concordant with this body of evidence: older age showed a borderline association with delirium, and the exploratory age threshold of approximately 71 years closely mirrors the 70-year cutoff highlighted by Wu et al., while transfusion—markedly less frequent after OLIF-PPSF—was associated with delirium in our cohort; operative time itself was not significantly associated with delirium in univariable analysis. Within the TLIF cohort, however, the association with transfusion disappeared after adjustment for age and era, suggesting that transfusion was largely a marker of the earlier treatment period rather than an independent precipitant. These null findings should be interpreted with caution: with only nine events in the matched cohort and sixteen in the eligible TLIF cohort, the study had limited power to detect associations of moderate size, and the strong collinearity between transfusion, blood loss, and calendar period further limits what can be inferred about their individual contributions. The association with age, although it did not reach statistical significance in the matched cohort, is consistent with established age thresholds and suggests that age remains the most dependable marker of risk in this population, in whom the consequences of delirium are also most severe. Preexisting dementia, a recognized risk factor for postoperative delirium [12,14], also showed an association with delirium in our cohort, although the small number of affected patients precluded a precise estimate; all delirium cases among patients with dementia occurred in the TLIF group. The 6.4% incidence in the TLIF group is consistent with the previously reported incidence of delirium after lumbar fusion (approximately 3–20%) [11,12].

Beyond these associations, we examined whether transfusion might lie on the pathway between the surgical approach and delirium. When transfusion was substituted for age in the multivariable model, the association of the TLIF approach with delirium was attenuated and transfusion was no longer independently significant. Attenuation of this kind does not by itself demonstrate mediation, however, and with only nine events formal estimation of direct and indirect effects was not feasible, so the pattern should be regarded as exploratory. It is equally compatible with transfusion acting as a marker of the earlier treatment era, an interpretation supported by the fall in the transfusion rate within the TLIF group from 79.6% in 2008–2011 to 20.6% in 2017–2023.

Postoperative delirium is not a benign event; it is associated with prolonged hospitalization, functional decline, falls, increased cost, and higher mortality [13,14]. Our data nevertheless do not support selecting one fusion technique over another for the purpose of preventing delirium, because the observed difference cannot be separated from the calendar period in which each procedure was performed. What the data do support is the established observation that older patients undergoing lumbar fusion are at increased risk, and that this risk should inform perioperative monitoring whichever approach is chosen. Patients at elevated baseline risk—those of advanced age, with multiple comorbidities, cognitive impairment, or a high anticipated transfusion requirement [12,37]—warrant particular attention.

This study also has several strengths. Propensity score matching on seven clinically relevant covariates produced well-balanced groups, with standardized mean differences below 0.20 for all matching covariates, allowing a fairer comparison of delirium incidence than would be possible in an unmatched retrospective cohort. Moreover, because postoperative delirium did not occur in the OLIF-PPSF group, conventional logistic regression would have produced unstable and uninterpretable estimates; the use of Firth’s penalized likelihood yielded valid effect estimates under this quasi-complete separation, adding methodological rigor to the risk-factor analysis. Finally, because all operations were performed by a single experienced surgeon, inter-surgeon variability was eliminated; this does not, however, eliminate confounding by indication, calendar time, the learning curve, or changes in perioperative care.

This study has several limitations. First, it is a retrospective study conducted at a single center and based on the experience of a single surgeon; although this minimized inter-surgeon and inter-institutional variability, the findings require validation in multicenter cohorts involving multiple surgeons. Moreover, because the surgical approach was selected at the surgeon’s discretion rather than at random, residual confounding by indication cannot be fully excluded despite propensity score matching on measured covariates. In particular, because TLIF was more often selected for pathology requiring direct decompression, the two groups may have differed in the type and severity of the underlying disease in ways not fully captured by the matched covariates, and such unmeasured differences could have contributed to the observed disparity in delirium. Second, and most importantly, postoperative delirium did not occur in the OLIF-PPSF group, resulting in only nine events overall; although Firth’s penalized regression provides valid estimates under this quasi-complete separation, the wide confidence intervals reflect limited precision, and the magnitude of the association should be interpreted cautiously. The present findings are therefore best regarded as hypothesis-generating. Third, delirium was diagnosed by psychiatric consultation prompted by clinical suspicion rather than by routine screening with a validated instrument such as the Confusion Assessment Method, which may underestimate the incidence of delirium, particularly the hypoactive subtype; the absence of diagnosed delirium in the OLIF-PPSF group should therefore not be taken as evidence that delirium does not occur after this procedure, and indeed, among patients excluded because they underwent combined procedures or surgery for infection, postoperative delirium did occur after OLIF-PPSF as it did after TLIF; although the same ascertainment process was applied throughout, we cannot exclude changes in recognition thresholds over a 16-year period, so the observed decline in diagnosed delirium may reflect changes in detection as well as in perioperative care. Fourth, and most importantly, the two procedures were performed in almost entirely different calendar periods: OLIF-PPSF was introduced only in 2017, and every delirium event occurred between 2008 and 2014, so the effect of the surgical approach cannot be separated from secular changes in anesthesia, transfusion practice, perioperative care, and delirium recognition. Finally, some baseline variables, including the JOA score and dementia, were not included as matching covariates; the former showed minor residual imbalance, although dementia—a recognized risk factor—was infrequent and well balanced between the matched groups. In addition, only patients treated with a single, uniform surgical approach at all operated levels were included, whereas in clinical practice different approaches are sometimes combined across segments, which may limit the generalizability of the findings. Variables such as cognitive status, frailty, American Society of Anesthesiologists physical status, stenosis severity, and cumulative opioid dose were not available; previous lumbar surgery was likewise not recorded. Hemoglobin concentrations, the number of red blood cell units transfused, the timing of transfusion, and the use of cell salvage or tranexamic acid could not be retrieved for the whole cohort, so a dose–response analysis was not possible, and opioid and benzodiazepine exposure was recorded only as a binary variable rather than as morphine-equivalent or cumulative dose. Patient-reported outcomes were compared at the last available visit, and the variable follow-up interval may have introduced additional heterogeneity; the matched-pair identifiers were not retained, so continuous variables were compared without accounting for the matched design [25]; and delirium was captured only during the index admission, so episodes occurring after discharge were not identified.

## 5. Conclusions

In this propensity score-matched analysis, the incidence of postoperative delirium was lower after OLIF-PPSF than after TLIF. Every event, however, occurred before OLIF-PPSF was introduced, and no delirium occurred after either procedure during the period in which both were performed; the difference therefore cannot be attributed to the surgical approach and more likely reflects changes in perioperative care over time. The parallel decline in the transfusion rate over the same period is consistent with this interpretation, and transfusion was associated with delirium in univariable analysis, although this association did not persist after adjustment for age and treatment era. Older age was the only factor consistently associated with delirium. A muscle-sparing approach that reduces blood loss and transfusion nevertheless remains biologically plausible as a means of lowering delirium risk, and this possibility—neither supported nor excluded by the present data—warrants prospective evaluation in adequately powered cohorts. These findings should be regarded as exploratory and underline the importance of accounting for calendar time when a newly adopted operation is compared with an established one; the absence of diagnosed delirium after OLIF-PPSF should not be read as proof that it does not occur.

## Figures and Tables

**Table 1:** Distribution of operations by calendar year.

	Eligible Cohort		Matched Cohort	
Year	OLIF-PPSF (*n* = 168)	TLIF (*n* = 319)	OLIF-PPSF (*n* = 141)	TLIF (*n* = 141)
2008	—	23	—	5
2009	—	27	—	5
2010	—	28	—	10
2011	—	30	—	12
2012	—	60	—	22
2013	—	42	—	21
2014	—	32	—	17
2015	—	16	—	8
2016	—	27	—	18
2017	16	13	12	8
2018	29	6	28	5
2019	34	4	28	3
2020	23	3	19	2
2021	26	6	22	4
2022	31	1	25	—
2023	9	1	7	1
Total	168	319	141	141

OLIF-PPSF was introduced in 2017. Before 2017 no OLIF-PPSF procedure was performed, and 118 of the 141 matched TLIF operations (83.7%) were performed during that earlier period.

**Table 2:** Baseline characteristics before and after propensity score matching.

	Before Matching			After Matching		
	OLIF-PPSF (*n* = 168)	TLIF (*n* = 319)	SMD	OLIF-PPSF (*n* = 141)	TLIF (*n* = 141)	SMD
Age (years)	69.5 ± 7.7	64.4 ± 9.8	**0.57**	68.5 ± 7.5	69.0 ± 7.3	−0.07
Sex (male)	58 (34.5%)	105 (32.9%)	0.03	44 (31.2%)	36 (25.5%)	0.13
Body mass index	24.7 ± 3.6	24.5 ± 3.1	0.06	25.0 ± 3.6	24.5 ± 3.2	0.13
Number of fused levels	1.46 ± 0.64	1.24 ± 0.47	**0.39**	1.39 ± 0.56	1.33 ± 0.54	0.12
Year of surgery	2019.9 ± 1.8	2012.7 ± 3.1	**2.86**	2019.8 ± 1.7	2013.7 ± 3.1	**2.46**
Operated levels (L1–2/L2–3/L3–4/L4–5/L5–S1)	2/15/87/138/3	6/11/70/207/100	–	0/11/72/111/1	4/6/38/90/49	–
**Diagnosis**						
Spinal stenosis	100 (59.5%)	195 (61.1%)	−0.03	84 (59.6%)	77 (54.6%)	0.10
Degenerative spondylolisthesis	68 (40.5%)	124 (38.9%)	0.03	57 (40.4%)	64 (45.4%)	−0.10
**Preoperative patient-reported scores**						
VAS (back)	5.6 ± 1.3	5.5 ± 0.9	0.12	5.5 ± 1.3	5.5 ± 0.7	−0.01
VAS (leg)	6.8 ± 1.2	6.7 ± 0.7	0.11	6.7 ± 1.2	6.7 ± 0.8	−0.01
JOA score	14.6 ± 5.2	16.0 ± 3.3	**−0.32**	14.6 ± 5.2	16.0 ± 3.7	**−0.31**
ODI	28.5 ± 7.3	27.2 ± 4.8	**0.22**	27.9 ± 7.1	28.1 ± 5.1	−0.04
**Comorbidity**						
Hypertension	106 (63.1%)	158 (49.7%)	**0.27**	84 (59.6%)	90 (63.8%)	−0.09
Diabetes mellitus	50 (29.8%)	75 (23.5%)	0.14	40 (28.4%)	36 (25.5%)	0.06
Hepatitis	5 (3.0%)	7 (2.2%)	0.05	4 (2.8%)	4 (2.8%)	0.00
Chronic kidney disease	12 (7.1%)	6 (1.9%)	**0.26**	5 (3.5%)	6 (4.3%)	−0.04
Cerebrovascular accident	13 (7.7%)	6 (1.9%)	**0.28**	3 (2.1%)	4 (2.8%)	−0.05
Dementia	6 (3.6%)	9 (2.8%)	0.04	5 (3.5%)	4 (2.8%)	0.04

Values are mean ± standard deviation or number (%). SMD, standardized mean difference; values in bold indicate |SMD| > 0.20. Year of surgery and diagnosis were not included in the propensity score model. The JOA score was likewise not a matching covariate. OLIF-PPSF, oblique lateral interbody fusion with percutaneous pedicle screw fixation; TLIF, transforaminal lumbar interbody fusion; VAS, visual analog scale; JOA, Japanese Orthopaedic Association score for low back pain (range −6 to 29; higher scores indicate better function); ODI, Oswestry Disability Index.

**Table 3:** Perioperative outcomes and postoperative delirium in the matched cohort.

	OLIF-PPSF (*n* = 141)	TLIF (*n* = 141)	*p*-Value
Operative time (min)	262.2 ± 75.5	280.9 ± 66.5	**0.028**
Estimated blood loss (mL)	283.3 ± 202.3	393.3 ± 241.1	**<0.001**
Transfusion	20 (14.2%)	96 (68.1%)	**<0.001**
Length of hospital stay (days)	12.3 ± 6.5	17.7 ± 8.5	**<0.001**
Postoperative delirium	0 (0%)	9 (6.4%)	**0.004**
Opioid use	63 (44.7%)	76 (54.0%)	0.122
Benzodiazepine use	2 (1.4%)	3 (2.1%)	1.000

Values are mean ± standard deviation or number (%). The comparison of delirium was made with McNemar’s exact test; the absolute risk difference was 6.4% (95% CI 2.4–11.7%), and Fisher’s exact test gave *p* = 0.003. Incidence was 0% (95% CI 0–2.6%) after OLIF-PPSF and 6.4% (95% CI 3.0–11.8%) after TLIF. Other categorical variables were compared with Fisher’s exact test and continuous variables with independent-sample *t*-tests. Opioid and benzodiazepine use denotes administration at least once during the postoperative hospital stay. Bold indicates *p* < 0.05.

**Table 4:** Postoperative delirium and transfusion by treatment era in the eligible cohort.

	TLIF			OLIF-PPSF		
Period	*n*	Delirium	Transfusion	*n*	Delirium	Transfusion
2008–2011	108	3 (2.8%)	86 (79.6%)	—	—	—
2012–2014	134	12 (9.0%)	103 (76.9%)	—	—	—
2015–2016	43	1 (2.3%)	25 (58.1%)	—	—	—
2017–2023	34	0 (0%)	7 (20.6%)	168	0 (0%)	27 (16.1%)

Values are number (%). OLIF-PPSF was introduced in 2017. Within the contemporaneous period (2017–2023) neither the transfusion rate (16.1% versus 20.6%, *p* = 0.615) nor the operative time (268 ± 83 versus 259 ± 56 min, *p* = 0.547) differed significantly between the two procedures.

**Table 5:** Patient-reported outcomes in the matched cohort.

	OLIF-PPSF (*n* = 141)	TLIF (*n* = 141)	*p*-Value
**Postoperative scores (at last follow-up)**			
VAS (back)	2.6 ± 1.3	2.6 ± 1.5	0.681
VAS (leg)	4.7 ± 1.7	4.6 ± 1.9	0.642
JOA score	17.7 ± 4.5	18.1 ± 4.3	0.446
ODI	19.4 ± 6.8	19.2 ± 6.1	0.795
**Improvement from baseline**			
VAS (back)	2.9 ± 1.5	2.9 ± 1.7	0.758
VAS (leg)	2.0 ± 2.1	2.1 ± 2.2	0.697
JOA score	3.1 ± 5.1	2.1 ± 4.5	0.065
ODI	8.5 ± 7.4	8.9 ± 6.9	0.639

Values are mean ± standard deviation. Improvement denotes the change between the preoperative assessment and the last follow-up (VAS and ODI, preoperative minus postoperative; JOA, postoperative minus preoperative). Preoperative scores are shown in Table 2. Groups were compared with independent-sample *t*-tests. VAS, visual analog scale; JOA, Japanese Orthopaedic Association score for low back pain; ODI, Oswestry Disability Index.

**Table 6:** Firth penalized logistic regression for postoperative delirium in the matched cohort.

	Univariable OR (95% CI)	*p*-Value	Multivariable aOR (95% CI)	*p*-Value
Group (TLIF vs. OLIF-PPSF)	20.30 (1.16–355.88)	**0.039**	19.82 (1.20–326.53)	**0.037**
Age (per year)	1.10 (0.99–1.21)	0.069	1.09 (0.99–1.21)	0.074
Sex (female)	0.48 (0.13–1.72)	0.259		
Number of fused levels	1.85 (0.69–4.98)	0.224		
Operative time (per min)	1.00 (0.99–1.01)	0.629		
Transfusion	4.52 (1.05–19.40)	**0.042**		
Hypertension	1.93 (0.45–8.30)	0.377		
Diabetes mellitus	2.25 (0.63–8.10)	0.214		
Hepatitis	1.63 (0.07–36.00)	0.757		
Chronic kidney disease	1.19 (0.06–24.62)	0.909		
Cerebrovascular accident	1.86 (0.08–42.39)	0.698		
Dementia	11.84 (2.22–63.22)	**0.004**		
Body mass index	0.98 (0.81–1.19)	0.850		

Estimates were obtained by Firth’s penalized logistic regression to address quasi-complete separation (no delirium in the OLIF-PPSF group). The multivariable model was restricted to group and age because only nine events occurred. Because the two procedures were performed in almost entirely different calendar periods, the group variable cannot be separated from the treatment era and should not be interpreted as an effect of the surgical approach. OR, odds ratio; aOR, adjusted odds ratio; CI, confidence interval. Bold indicates *p* < 0.05.

**Table 7:** Exploratory model including transfusion in place of age.

	aOR (95% CI)	*p*-Value
Group (TLIF vs. OLIF-PPSF)	16.34 (0.89–301.60)	0.060
Transfusion	1.49 (0.35–6.45)	0.590

Firth penalized logistic regression in the matched cohort. Because transfusion lies on the causal pathway between the surgical approach and delirium, this model is reported as exploratory; attenuation of the group estimate after adding transfusion does not by itself demonstrate mediation, and formal estimation of direct and indirect effects was not feasible with nine events. aOR, adjusted odds ratio; CI, confidence interval.

## Data Availability

The data presented in this study are available on reasonable request from the corresponding author. The data are not publicly available owing to privacy and ethical restrictions.
